# A mixed methods evaluation of time management products for persons with dementia in India: what works, what does not, and what may

**DOI:** 10.1186/s12877-026-06985-y

**Published:** 2026-01-28

**Authors:** Sebestina Anita Dsouza, Kshama Bangera, Vinita Acharya, Vasudeva Guddattu

**Affiliations:** 1 Department of Occupational Therapy, Manipal College of Health Professions, Manipal Academy of Higher Education, Manipal, Karnataka India; 2 Centre for Studies on Healthy Aging, Manipal Academy of Higher Education, Manipal, Karnataka India; 3 Department of Data Science, Prasanna School of Public Health, Manipal Academy of Higher Education, Manipal, Karnataka India

**Keywords:** Assistive technology, Daily time management, Dementia, Caregiving, Participation, Well-being

## Abstract

**Background:**

Persons with dementia (PwD) experience difficulties in daily time management (DTM), which restricts their participation in valued activities. Time management products (TMPs) are assistive devices that support DTM and functional independence. Most previous research on TMP has been based on developed regions of the world. Given the limited research in low- and middle-income countries, the present study aimed to explore the use of TMP by PwD and caregivers in India.

**Methods:**

We conducted a mixed-methods, explanatory sequential study. We first undertook a single-group, prospective, prepost, interventional study involving 38 dyads of persons with mild to moderate dementia and their caregivers. We assessed the self-reported and caregiver-reported DTM, performance and satisfaction in valued daily activities, time processing abilities, and well-being of the PwD and the caregivers’ well-being and ability to cope. The participants were then encouraged to use the TMP provided as an intervention. We reassessed the participants after three months and conducted semistructured interviews with three PwD and 12 caregivers to understand their experience of using the TMP.

**Results:**

Within-group analysis revealed minimal but statistically insignificant changes in the scores of the PwD and caregiver on all the assessments. These findings suggest that TMP may help maintain the ability of PwD, especially those with mild dementia, and support caregivers to some extent. Thematic analysis revealed benefits to PwD, including reduced annoyance with caregivers’ prompts, improved confidence in knowing the time and doing valued activities more independently, increased caregiver involvement in supporting device use, complex operation of some products, and suggestions to make the products more suitable for use in India. The study also identified salient factors that may influence the use of TMPs, including PwD and caregivers’ need and priority for DTM; personal characteristics; and contextual factors, especially living arrangements and prevalent sociocultural attitudes towards time, devices, and elders. An increased demand for such products in the future is also anticipated.

**Conclusion:**

This study provides encouraging evidence on the usefulness and acceptance of TMP by PwD and caregivers in India, although DTM may not be a priority for most individuals. The adoption of such products would entail a person-centred, context-specific approach to the development and provision of assistive technology.

**Trial registration:**

The trial was registered with the Clinical Trail Registry - India (CTRI/2017/06/008916) on 27 June 2017.

## Background

Dementia is a progressive debilitating neurological disorder affecting global cognitive ability. Persons with dementia (PwD) experience time-related difficulties such as poor time awareness, disorientation, and poor time perception and estimation [1–5]. These time-related difficulties are attributed to impaired cognitive functions, including time-related cognitive functions. These abilities are also referred to as time-processing abilities (TPA) and entail time orientation, perception, and management [6, 7]. Owing to impaired TPA, PwD experience difficulties in managing their time for daily activities or daily time management (DTM) [8]– [9]. For example, PwD need more time for daily activities, have difficulty remembering and planning what to do, when, and for how long, following routines, and adapting to time demands. As dementia progresses, PwD require the assistance of caregivers for their DTM [10–13]. 

To support PwD in their daily activities, occupational therapists commonly prescribe assistive technologies (ATs) [14]. Time management products (TMPs) are assistive devices that support the TPA and DTM [15]. These range from simple (e.g., night-day calendars) to advanced (e.g., smartphones, electronic calendars, digital planning boards, and robots) products or devices. Studies in developed countries suggest benefits with TMP, including improved orientation, reduced effort, worry and reliance on caregivers, improved sense of control of PwD and well-being of PwD and caregivers [12, 16–29]. The studies were predominantly based in Europe, Ireland and the United Kingdom and included PwD living at home and long-term settings [12, 16–29]. These studies have also reported factors influencing the adoption of TMP, including PwDs’ attitudes towards time, perceived need, awareness and severity of cognitive impairments, disease progression, and living situation (alone or with family); caregivers’ motivation and time to support PwD in using TMP; and product-related features such as size, display, portability, ease of use, and cost [12, 16–29].

By 2050, two-thirds of the global population aged 60 years and above will be in low- and middle-income countries (LMICs) [30]. India, a LMIC is witnessing an increasing prevalence of dementia [31, 32] alongside an aging population [33]. The inequalities in access to rehabilitation services among developed and developing regions worldwide highlight the need to support the use of AT in LMICs. There is limited research in LMICs on AT that addresses cognitive impairments and the needs of the aging population, especially PwD [15, 34, 35]. In view of this research and practice gap, we conducted a qualitative study to understand the current practices of Indian occupational therapists and caregivers for supporting the DTM of PwD and their expectations of TMP [36]. The findings suggest that caregivers address cognitive deficits in attention, orientation, and memory with simple products (diaries, alarms, schedules) and do not prioritize DTM. These practices are attributed largely to contextual factors, including polychronic attitudes towards time where doing things on time is not a priority, the collective culture that values interdependence, negative societal attitudes towards elders and people with disabilities, poor knowledge about dementia, impairment-oriented interventions, and limited access to AT. Devices to support DTM, if needed, are expected to be low-cost, simple, portable, customizable, and usable across the disease continuum [36]. The present paper presents the findings of a subsequent interventional study undertaken to explore the use of TMP in the Indian context. The objectives of the study were as follows:

1. To investigate the impact of TMP on the daily time management and well-being of PwD and their caregivers.
2. To understand the experiences of PwD and their caregivers in using TMP.

## Methods

### Study design

The study used a mixed-methods, explanatory sequential design. Objective 1: This was a single-group, prospective, pre-post, interventional study to understand the impact of TMP on PwD and their caregivers. This design was deemed suitable in the absence of any prior research on TMP in India. Objective 2 entailed a qualitative descriptive approach to understand the participants’ experiences of using TMP. The researchers used straightforward language, presented direct descriptions of the participants’ narratives, and attempted to be as close to the data as possible [37].

### Study site

The study was conducted in urban and semi-urban areas of Udupi and Mangalore districts, Karnataka, South India. Dementia care in these settings emphasizes medical management available at hospitals or clinics. Rehabilitation services, including occupational therapy, are limited [36, 38, 39]. Kannada (official regional language of the state), Konkani, Tulu and English are the languages spoken in the region.

### Sample size and participant recruitment

The study participants were dyads of PwD and caregivers. We used the formula for the difference between two means to calculate the sample size [40]. In the absence of literature for values of standard deviation (σ) and clinically significant difference (d) in the formula, we assumed that σ = 1 and d = 0.5 for the primary outcome measure of TPA on the basis of our clinical experience. With 80% power, a 5% level of significance, and a 20% dropout rate, a sample size of 38 dyads was calculated for the study, i.e., 38 PwD and 38 caregivers. The participants were recruited from hospitals, clinics and senior organizations in the study setting. To facilitate recruitment, we contacted the doctors and authorities of organizations for referrals and advertised the study details in local newspapers and social media. PwD and caregiver dyads meeting the study criteria were included in the pre-post study via convenience sampling. The participants for the qualitative study were purposively selected from the pre-post study on the basis of the devices provided, living arrangement, and ability to participate in an interview.

Inclusion and exclusion criteria.

The study included PwD aged 60 years or older diagnosed with dementia with self-reported or caregiver-reported difficulties in DTM at the time of screening, who were able to understand and communicate in English or Kannada, and who were willing to try the TMP for three months. PwD with severe dementia (Clinical Dementia Rating Scale score of 3) [41], psychiatric disorders unrelated to dementia, high physical frailty, and poor verbal communication were excluded. The study included caregivers able to understand and communicate in English or Kannada who identified themselves as primary carers of the PwD and were familiar with the PwD’s routines.

### Outcome measures

PWD and caregivers’ age, gender, relationship, education, employment, living arrangement, and details of caregiving responsibilities were collected. The following assessments were administered at pretest and posttest after three months:

1. Kit for Time-processing Ability-Senior (KaTid-Senior): A therapist-administered 29-item instrument to assess TPA, including time perception, orientation, and management, in older adults. Higher values indicate better TPA [13, 42, 43].
2. Time-Self Rating Senior (Time-S-Senior): A self-rated 21-item instrument for DTM. Higher scores indicate better daily time management [13, 42, 43].
3. Time-Proxy Senior (Time-P-Senior): A 13-item instrument to obtain the caregiver’s report of the PwD’s DTM and includes 13 items. Higher scores indicate better DTM [42, 43].
4. Canadian Occupational Performance Measure (COPM): a self-report questionnaire to identify and measure performance and satisfaction in prioritized activities. Higher scores indicate higher performance, satisfaction, and priority [44].
5. World Health Organization-5 Well-being Index (WHO-5): A 5-item scale to assess psychological health over the previous two weeks. Higher total scores suggest better well-being [45, 46]. The PwD and caregiver completed the questionnaire individually.
6. Carers of Older People in Europe (COPE) Index: A 15-item instrument measuring the negative impact, positive value and quality of support as perceived by caregivers. Higher scores on the negative impact component indicate greater stress. Lower scores on the positive value and quality of support component indicate lower caregiver satisfaction and support [47–49].

These assessments were adapted to the Indian context and/or translated into the Kannada language with permission from developers via recommended guidelines [50], validated with experts and tested with ten older adults. The process is described in another study [13].

For the qualitative study, separate interview guides were developed for PwD and caregivers. The interview guides were developed in English on the basis of the findings of our previous study [36]. The key questions for the caregivers were as follows: (1) *Could you describe your experience of using the TMP provided to your family member with dementia?* (2) *On the basis of your experience*,* what are your suggestions for using these devices in the Indian context*? The interview questions progressed from broad to specific and included open-ended questions with probes such as “Could you elaborate?” or “Could you explain with an example…?” for elaboration and clarification. The interview guide for PwD used short simple questions to encourage participation. The interview guides were translated into the Kannada language, piloted and revised. The pilot interview is included in the study.

### Intervention

TMPs are not readily available in India. For the study, we imported five TMPs and used two local devices as follows:

- An electronic calendar, a senior-friendly, easy-to-use and read clock, displays the day, date, time, and part of the day (morning, afternoon, evening or night).
- An electronic calendar with alarms/voice messages for reminders.
- A timer with options to set the duration of an activity for 15, 30, 45 and 60 min.
- An electronic planner, an advanced device for time management, provides a visual overview of the day with alarms for reminders. It has a magnetic whiteboard for writing and uses magnetic activity picture cards or sticky notes. Time is represented in a linear format with small lights for every quarter (15 min) that go off as time passes.
- A planning aid, an advanced internet-enabled tab-based electronic device for time management, supports a visual overview of the day with pictorial representations of time and activities, reminders that could be personalized, and remote access.
- A pictorial planner, developed for the project using locally available magnetic whiteboards with pictorial time slots for parts of the day and provided with a whiteboard marker pen and/or magnetic pictorial activity cards tailored to the participant’s needs.
- A locally available standard digital clock.

One or more devices were provided on the basis of the pretest TPA and DTM scores and the priorities and preferences of PwD and caregivers [36]. For example, advanced TMPs were provided to PwDs with caregivers familiar with technology. The electronic calendar or digital clock was provided for time orientation singly or with the pictorial-planner or electronic planner for the DTM. A timer was provided for PwD experiencing difficulties in judging the duration of activities such as bathing or going for a walk.

### Procedure

The study was conducted between 16 September 2017 and 31 July 2022. Potential participants were identified through the study’s recruitment strategies. KB briefed them about the study over the phone or in person. Interested participants were screened at their residence or hospital/clinic. Dyads meeting the study criteria and providing informed (verbal and written) consent were included and provided with the participant information sheet. KB then visited the dyads at their home on the basis of their convenience for the pretest. The participants were given rest intervals as and when needed. KB then provided the TMP with instructions and demonstrations along with a handout and device user manual. They were contacted regularly to provide support and encourage adherence. The participants were reassessed after three months. Feedback about the TMP was also noted. All assessments and interventions were performed in a quiet distraction-free environment in the participant’s preferred language over one or two sessions.

In addition, the participants selected for the qualitative study were interviewed in person or via telephone at posttest. Efforts were made to avoid leading questions or comments and agree or disagree with the participants. All interviews were audio-recorded and ranged from 20 to 52 min, with an average duration of 37 min. KB made notes during and immediately after the interview. KB transcribed the audio recordings verbatim within two to three days. The transcripts were anonymized to maintain the confidentiality of the participants. All the investigators were proficient in English and Kannada. KB translated the interviews in the Kannada language into English with care to ensure semantic accuracy [51]. The coinvestigators verified the transcribed data with the recordings to ensure accuracy. Bilingual experts were consulted for translation. The back translations were performed by an expert. Another expert verified the translations. The deidentified transcripts were then printed. The study was suspended during the COVID-19 pandemic because of the imposed lockdown. When restrictions were lifted, data collection was resumed with recommended guidelines and precautions. KB collected most of the data online or via telephone. In-person visits were restricted to assessing the PwD (KaTid-Senior and Time-S-Senior) and providing the devices. The interviews were stopped when data saturation was reached.

### Data analysis

Quantitative analysis was done using Jamovi software (version 1.6.23, solid) [52]. Descriptive statistics were computed for participant characteristics. The data were normally distributed. Within-group analysis was performed with the paired t-test for the total sample. All outcome measures showed similar trends at posttest, except for the Time‒S‒Senior score. Therefore, subgroup analysis was performed for PwD with mild to moderate dementia. The level of significance was *p* < 0.05.

The qualitative data were analysed manually via thematic analysis [53]. Analysis commenced with the initial interview. In the initial step, all the investigators read the transcribed interviews multiple times to familiarize themselves with the data, referred to the field notes and independently conducted an initial round of coding. Codes that exhibited commonalities were grouped into categories and themes. The preliminary categories and themes underwent an iterative process of review and refinement to ensure that they accurately captured the participants’ experiences and were aligned with the study’s objectives. The investigators resolved disagreements through discussions. SD and VA were experienced with qualitative research. KB received training for qualitative research and conducted practice interviews under supervision. The investigators lived in the study setting and were familiar with the language, culture, and practice context. To reduce bias, the investigators individually read and coded the transcripts, referred to the field notes, questioned their assumptions, continually engaged in reflexivity, and met regularly to discuss their interpretations. SD and VA are occupational therapists with extensive experience in neurorehabilitation and mental health, respectively, whereas KB is a social worker experienced in working with older adults. They brought different perspectives that contributed to the rigor of the study.

## Results

We identified 64 dyads, 38 of which met the study selection criteria and consented to participate. The study included 17 participants (44.7%) with mild dementia and 21 (55.3%) with moderate dementia. The participant characteristics are summarized in Table 1. The mean ages of the PwD and caregivers were 76.0 ± 6.79 years and 54.3 ± 14.8 years, respectively. Most caregivers were adult children or spouses and lived with the PwD. At the time of recruitment, among the PWD, seven women were still involved in household chores, and three men continued to participate in their businesses with the support of family members.

**Table 1:** Characteristics of participants of the pre-post study

Characteristics		Person with dementia (*N* = 38)	Caregiver (*N* = 38)
Gender	Male	18 (47.4%)	16 (42.1)
	Female	20 (52.6%)	22 (57.9%)
Education	Primary school	8 (21.05%)	0
	Secondary school	9 (23.68%)	7 (18.4%)
	Graduate and above	20 (52.6%)	31 (81.6%)
Marital status	Married	20 (52.6%)	31 (81.6%)
	Divorced/widowed/single	18 (47.4%)	7 (18.4%)
Occupations	Full-time employed or self-employed	11 (28.9%)	21 (55.3%)
	Part-time employed and/or self-employed	0	2 (5.3%)
	Retired	12 (31.5%)	9 (23.7%)
	Homemaker	15 (39.5%)	6 (15.8%)
Income meets needs adequately	No	-	18 (47.36%)
	Yes	-	20 (52.63%)
Relationship to person with dementia	Spouse	-	11 (28.9%)
	Sibling	-	3 (7.9%)
	Child	-	15 (39.5%)
	Daughter/son-in-law	-	4 (10.5%)
	Others (extended family/friends)	-	5 (13.15%)
Living arrangement	In the same household	-	32 (84.2%)
	Different household but in the same building	-	1 (2.6%)
	Living nearby	-	5 (13.1%)
Providing care to anyone else	No	-	24(63.16%)
	Yes	-	14 (36.84%)
Caregiving days/week	Four to six days/week	-	2(5.3%)
	Daily	-	36(94.7%)

At posttest, five PwD (13.15%) and three caregivers (7.89%) withdrew from the study because of an acute medical illness. Finally, 33 PwD (16 with mild dementia, 17 with moderate dementia) and 35 caregivers (16 caregivers of PwD with mild dementia, 19 caregivers of PwD with moderate dementia) completed the posttest. The overall dropout rate of the study was 6.57%. As shown in Table 2, at posttest, the PwD’s scores on the KaTid-Senior, Time-P-Senior, WHO-5 and COPM improved marginally, although statistically insignificantly, but the Time-S-Senior scores decreased significantly (*p* < 0.05). The caregiver scores on the WHO-5 and COPE Index scores decreased marginally (statistically insignificantly) at posttest. The significant reduction in Time-S-Senior scores was not consistent with other assessments, especially Time-P-Senior. To assess this, subgroup analysis was performed. The findings were similar to the results of the total sample for all outcome measures, except for Time-S-Senior, where participants with mild dementia presented minimal but insignificant decreases, *t*(15) = 0.63, *p* = 0.54, from pretest (58.63 ± 12.41) to posttest (57 ± 12.39), whereas PwD with moderate dementia presented a significant reduction, *t*(16) = 5.333, *p* < 0.001, from pretest (43.06 ± 8.24) to posttest (36.71 ± 7.48). Overall, the analysis suggests a small to medium effect of TMP on the outcome measures.

**Table 2:** Comparison of pre-test and post-test scores of study outcome measures

Outcome measure	Pre-test Mean (SD)	Post-test Mean (SD)	t-statistics	Mean difference	95% Confidence interval		*P*-value
					Lower	Upper	
**PwD** (*N* = 33)							
KaTid-Senior	18.21(5.69)	18.18(6.64)	0.03	0.03	−1.927	1.9875	0.975
Time-S-Senior	50.61 (12.99)	46.55 (14.35)	2.81	4.06	1.119	7.0019	0.008*
WHO-5	15.73 (4.64)	17.55 (4.31)	−1.99	−1.81	−3.676	0.0401	0.055
COPM-Performance	5.62 (1.47)	6.03 (1.76)	−1.45	−0.40	−0.981	0.1628	0.155
COPM-Satisfaction	5.21 (1.83)	5.89 (2.04)	−1.86	− 0.68	−1.425	0.0609	0.071
**Caregiver** (*N* = 35)							
Time-P-Senior	30.63 (7.76)	29.86 (8.16)	0.92	0.77	−0.920	2.4627	0.360
WHO-5	18.09 (4.15)	17.31 (4.77)	0.87	0.77	−1.027	2.5701	0.390
COPE-Negative impact	11.83 (3.14)	11.63 (2.93)	0.50	0.20	−0.611	1.0112	0.620
COPE-Positive value	13.40 (1.70)	13.37 (2.10)	0.07	0.02	−0.742	0.7989	0.940
COPE-Quality of support	12.77 (2.53)	12.17 (2.67)	1.42	0.60	−0.259	1.4586	0.165

* Significant at *p* < 0.05

**Fig. 1:**
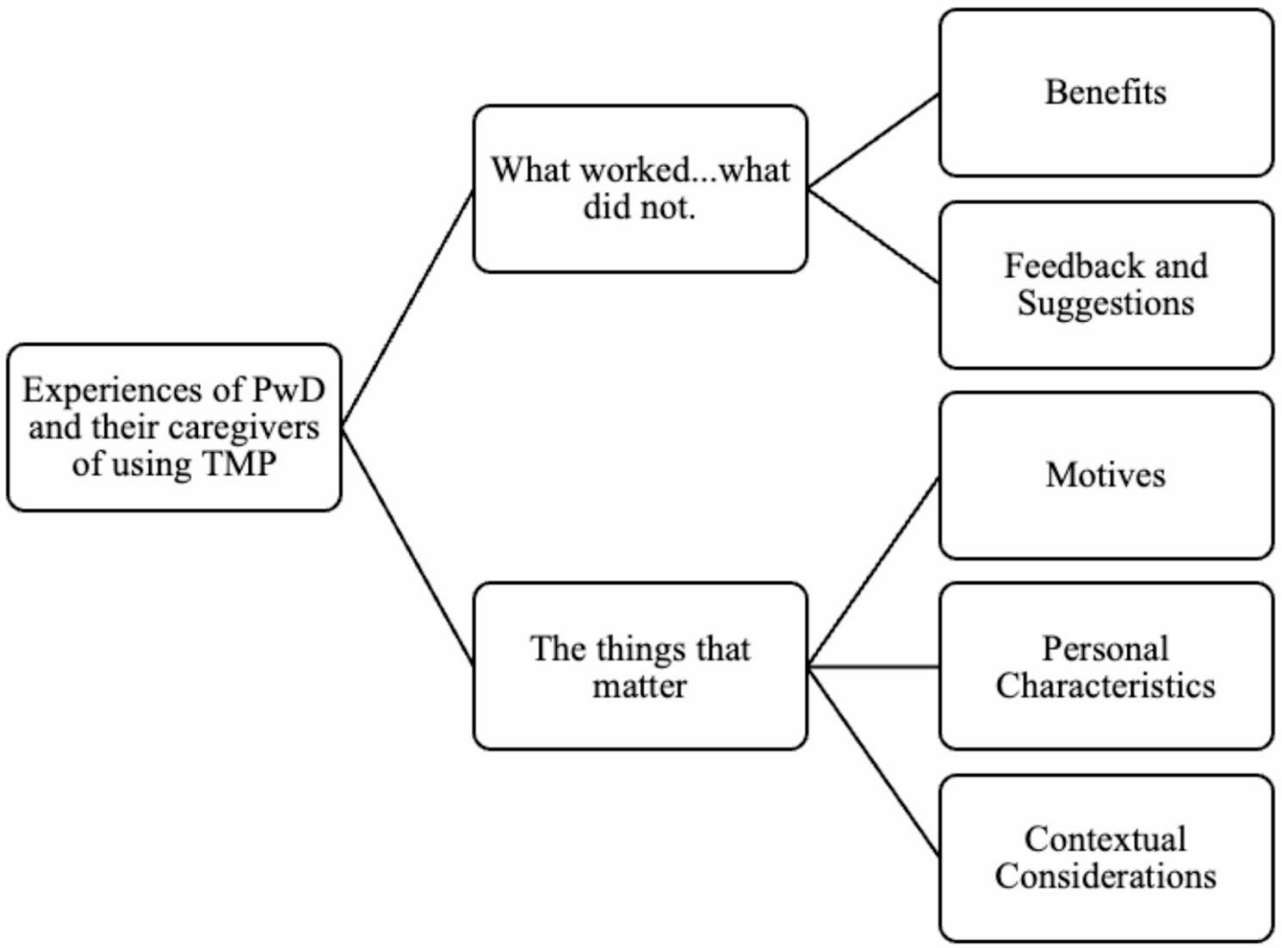
Thematic map

Three PwD and 12 caregivers participated in the qualitative study, which included one dyad. Their characteristics are summarized in Table 3. While all the PwD were engaged in self-care with caregivers’ support, four women and two men were similarly involved in household chores and business, respectively. Five caregivers had additional caregiving responsibilities. Five caregivers were financially comfortable, while others reported inadequate income. Eleven interviews (one PwD and ten caregivers) were in English, and four interviews (two PwD and two caregivers) were in Kannada. Twelve interviews were conducted in person, and three interviews were conducted via telephone. Thematic analysis identified two themes, which are illustrated in Fig. 1.

**Table 3:** Participant characteristics of qualitative study

Caregiver						Person with dementia							
ID	Age (years)	Education	Occupation	Relation to PwD	Lives with PwD	ID	Age (years)	Gender	Education	Occupation	Stage of dementia	TMP	Family
CG001	33	Graduate	Insurance Agent	Son	Yes		68	F	Primary	Homemaker	Moderate	EC, PP	Joint
CG002	34	Graduate	Lawyer	Son	Yes		74	M	Secondary	Business	Moderate	EC, T, EP	Joint
CG003	56	Graduate	Business	Wife	Yes		60	M	Graduate	Business	Moderate	EC, PP	Nuclear
CG004	72	Graduate	Retired Engineer	Relative	No		82	F	Graduate	Homemaker	Mild	EC, EP	Nuclear
CG005	41	Graduate	Medical Salesman	Son	Yes		79	M	Graduate	Retired Officer	Mild	EC, PP	Joint
CG006	70	Graduate	Retired Professor	Wife	Yes		75	M	Graduate	Retired Professor	Mild	EC, PP	Nuclear
CG007	28	Graduate	Lecturer	Son	Yes		73	M	Secondary	Business	Mild	PA	Nuclear
CG008	41	Graduate	Associate Professor	Son	No		83	M	Graduate	Retired Employee	Mild	EC, PP	Nuclear
CG009	42	Graduate	Business	Daughter	Yes	P002	65	F	Secondary	Homemaker	Mild	EC, PP	Joint
CG01	71	Graduate	Business	Husband	Yes	P003	66	F	Graduate	Homemaker	Mild	EC, PP	Nuclear
CG011	51	Graduate	Consultant	Son	No		86	F	Graduate	Retired Teacher	Mild	EC, PP	Nuclear
CG012	62	Graduate	Retired Accountant	Son	Yes		88	F	Graduate	Retired Teacher	Moderate	EC	Joint
	63	Graduate	Retired Engineer	Son-in-law	Yes	P001	87	M	Graduate	Retired Officer	Mild	ECR	Nuclear

*F *Female, *M* Male, *EC* Electronic Calendar, *ECR *Electronic calendar with reminders, *EP* Electronic Planner, *PA* Planning Aid, *PP* Pictorial Planner, *T* Timer

### Theme 1: what worked…what did not

This theme describes the participants’ experiences of using TMP and includes two categories: benefits and feedback and suggestions. Table 4 provides sample quotes for this theme.

**Table 4:** Theme 1: what worked…what did not

Categories	Codes	Sample quotes
Benefits	Devices easy to learn, time-related information in one place, having features for customization, supported independence and confidence in daily time management, reduced prompts and annoyance associated with reminders from caregivers, benefited the entire family	*It (Electronic calendar with reminders) speaks out loud and it is not a human. So*,* there’s no problem. Otherwise*,* we will get angry. Why are they saying the same thing so many times? No? Then I would say*,* “Why are you repeating the same*,* I will wake up.” With this (device)*,* I have to wake up. [P001]* *Since it’s a digital clock (Electronic calendar)*,* it’s easier to read. I would say it probably did help…at least partially to probably give her more sense of confidence that she still knows what time it is*,* even though she cannot read the hands of the analog clock. [CG011]* *That routine has been developed in the couple of days because of the board (electronic planner). Even with the timings it’s there. Whenever he wants to see the time and if we tell him to see*,* then he knows that it’s time to go to the hotel or it’s time to sleep. So*,* it’s useful…Like today if there is a specific function also*,* we write in bold letter*,* colour it or draw some pictures*,* so there is some attraction*,* he will look at it and he knows that today we have to go somewhere and there is some function there. So*,* we keep repeating. And whenever he is late to take a shower or have breakfast*,* we tell him to look at the time and mention that we have to go there. So*,* he does his work faster. [CG002]* *We’ve recorded our voices (in the planning aid). So*,* this was a new thing*,* and it also attracted my dad so that*,* uh*,* he could follow*,* uh*,* scheduling and all. [CG007]*
Feedback and suggestions	Positives, concerns such as technical issues, complexity of setting up and operation, power supply, caregiver support required for device use, suggestions to make devices suitable to older adults and relevant to the Indian context	*Connecting it to the plug became difficult. X’s (other family member) mobile also had to be charged there. If it’s disconnected and then connected again*,* it doesn’t switch on soon. [PwD2]* *The negative would be that it required me to constantly inform that device was there. [CG011]* *In that board (electronic planner)*,* it would have been better if they had mentioned AM and PM. It’s a bit confusing. 1–12*,* he doesn’t know whether it is timing or a number and whether it is indicating a timing. It should be written like 1 AM*,* 2 AM*,* 3 AM etc. So that he is aware that this is morning or evening etc. That should be there. Nothing more. [CG002]* *Yeah*,* there was a situation*,* but then my son came and then he rectified it [CG003]* *The setting part and all was slightly complicated. Because the thing is a new thing… It was confusing information that settings*,* how to do*,* and all. X (research assistant) guided me. Also*,* using the manual was helpful. [CG007]* *Support*,* yes. But this device (planning aid) must be kept in a particular place. So*,* if a person is residing in a particular place*,* like at home only*,* then it’s okay. But*,* uh*,* if a person’s traveling or some other activity he’s doing*,* he cannot carry this device always. So that then the*,* the variation in this*,* like usage of this can affect him. It cannot be like*,* because*,* uh*,* it’s*,* the device is a little bit heavy. [CG007]*
		*Maybe in the device itself (electronic calendar)*,* if things like brushing and coffee in the morning*,* things that need to be done in the afternoon*,* things to be done in the evening and night are shown*,* then it’s good. All this should be in one device itself. Otherwise*,* now*,* we have to show the board (pictorial planner) separately. If it is on one device itself*,* it is nice… The size will have to be bigger. Or a button could be added to it where you could keep going to the next activity. [CG001]* *Yes. I will suggest that it should not be dependent on electricity considering the Indian scenario*,* with the disconnection of electricity being frequent. Battery based would be better*,* even if it’s a single or double A battery or whatever. Not the rechargeable*,* that would increase the cost. [CG011]* *I think with digital displays*,* one needs to consider the contrast*,* because when we are looking at elderly and their visual activity. I would consider that a very light background with dark letters would be better than just on a clean screen because at certain angles*,* they are just somehow visible. [CG011]* *Yeah*,* I think there’s a lot of work that needs to be done in terms of the use of local language (electronic calendar). So*,* the communication strategy is number one. The use of images (in pictorial planner) is helpful*,* definitely. But I think there needs to be more varied images. We need to probably look into some more images that reflect something more practical to the Indian context…The roll-up whiteboards that can be hung anywhere would be more appropriate than a cumbersome whiteboard. [CG011]* *Uh*,* yeah*,* one issue I’m having like*,* uh*,* the volume*,* if it’s still larger than it is better. Okay. So*,* like normally the elderly people*,* because of the age issues*,* may not hear it (the reminder). So slightly higher volume if it’s there than well. [CG007]* *Once dementia is advancing*,* the board (electronic planner)… I don’t feel you can recommend it. But watch (electronic calendar)*,* yes. [CG004]*

### Benefits

Caregivers expected that the TMP provided in the project would support DTM of PwD, reduce cues or reminders and improve their daily routines. The participants found the TMP helpful to some extent. Caregivers noted that PwD felt more confident about knowing the time, doing daily activities and following routines more independently. The devices reduced caregiver strain to some extent. A caregiver also reported that the entire family used the device, which further supported the PwD in using the device and following a routine. A PwD reported that the device reduced feelings of annoyance with caregivers.

*It (electronic calendar) speaks out loudly*,* and it is not human. So*,* there is no problem. Otherwise*,* we will get angry. Why are they (family members) saying the same thing so many times? Then*,* I would say*,* “Why are you repeating the same*,* I will wake up.” With this device*,* I must wake up. [P001]*

### Feedback and suggestions

The participants identified several positive and negative effects of the TMP provided. PwD were not interested in using timers, although these timers were simple to use and carry around. PwD had difficulty reading the visual display of the digital clock because of the segmented digits, type of font, glare, and poor contrast. Both PwD and caregivers appreciated the personalization offered with voice message options in the electronic calendar with reminders and planning aids. The advanced devices were appreciated for being comprehensive in supporting orientation, what to do and when to do. However, caregivers also reported that PwD had difficulty understanding the concept of time in a linear format and the passage of time with small lights. The caregivers also found these devices complex to operate or manage, and some older caregivers required the help of family members. The electronic devices must be plugged continuously. This posed a challenge because of the limited number of plug points, multiple users, and frequent power cuts. This also limited device placement to locations that were not easily accessible to the PwD, thereby limiting its use. The larger wall-mounted devices were not easily portable (electronic planners, pictorial planners and planning aids), which posed an issue if the PwD had to change residences or travel. The pictorial planner was reported as childish and silly and raised questions from visitors. The caregivers required considerable time and effort to encourage PwD to use the devices in general.

The participants also provided several suggestions to make the devices relevant to the Indian context. One common suggestion is to have battery-operated devices address frequent power cuts and improve portability and placement options. They also suggested having A.M./P.M. format followed in India, uses Indian languages for visual and auditory output, including accents and pictures reflecting Indian elders and activities. The devices should also consider aging-related issues, such as the size, glare, contrast and digits of the visual display and higher volume for the auditory output. Some caregivers suggested that advanced devices (electronic planners, planning aids) should be provided in the early stages of dementia. They also desired customization features such as reminders in the caregiver’s voice and personalized pictures. Among all the devices, the easy-to-use, senior-friendly electronic calendar was appreciated for its simplicity, size and user friendliness. The participants recommended adding features to support routines through the display of daily activities and personalized reminders in the electronic calendar.

*Now*,* we have to show the board [pictorial planner] separately. If it is on one device itself [electronic calendar]*,* it is nice… The size will have to be larger. Or a button could be added to it where you could keep going to the next activity. [CG001]*

### Theme 2: the things that matter

This theme describes salient factors to be considered for successful adoption of TMP in the Indian context. It includes three interrelated but distinct categories: motives, personal characteristics and contextual considerations. Table 5 provides sample quotes for this theme.

**Table 5:** Theme 2: the things that matter

Categories	Codes	Sample quotes
Motives	Need/no need to support memory, DTM, routines, functional independence, confidence and well-being of PwD, reduce caregiver strain	*She was looking at calendar*,* reading newspaper*,* memorizing the date*,* day and all. She kept looking at Facebook posts. Whenever she saw the post*,* she noted down the time*,* date*,* and day… She also uses the mobile alarm. So*,* she took little time to get adjusted to your device. Whatever you gave…Maybe mama was finding it difficult to use. Initially*,* it was interesting for her. But slowly she realized that she could do it on her own. So maybe she… she was not dependent on the device. [CG009]* *Uh*,* something*,* it was a new thing. Um*,* new technology. So yeah*,* it was*,* uh*,* I was initially motivated because of something new. Also*,* it’s free of cost. [CG007]* *As the care provider I would say that my focus has been to provide any sort of support… If there’s any technology that can probably be given*,* then so be it. I think it’s in that perspective I’m quite motivated to see if it helps. [CG011]* *In the morning*,* we use the radio up until eight o’clock. We know the time without using the clock. Later*,* I go to office. She is busy with household work…Time and all she is not following. No need also. I will tell you again…She’s not dependent on time. She’s purely a homemaker. And she’s not having any priority. [CG010]* *Yes*,* I would buy it. I got to know about these after you gave it. If it was available and she needed it*,* then I would buy it. One reason is that it could improve her memory. If she keeps looking at it*,* then she would follow it correctly. It is not for our sake or so. If she improves*,* it is possible to go out. Or else the problem is that along with other things we will have to look out for her too. It will be difficult…But the thing is that even I am not able to make time for it*,* both in the morning and evening. If we had kept doing it*,* she would have improved. [CG001]* *Since the lockdown*,* all his routine has completely changed and most of the time*,* he is idle not knowing what to do. That had a negative effect on him. [CG003]*
Personal Characteristics	Personality traits, familiarity with technology, stage of disease progression, comorbidities, ability for new learning (age or disease–related) personal preferences, personal constraints	*May be because of personality and because she has tune her to a certain routine*,* she feels why should I look at the board (electronic planner)*,* there is no need. [CG004]* *I am weak when it comes to technology. I don’t know much… I don’t know about the new methods. I have been using the mobile phone*,* but the touch screen is new. [CG006]* *Main thing is his nature is such a way and one thing is it is a new device…touch screen and all such things. He is not used to it. As I said he is using feature phones. Whenever a new thing comes*,* he just resists. Like why* we *require it or why we want it? Simply it is a waste of time. [CG007]* *Okay*,* so time management is very subjective*,* especially in the elderly. And that subjectivity is driven more by their ability and mobility. [CG011]* *Uh*,* since she was using digital (clock) since*,* uh*,* three*,* four years. Mm now she’s perfectly adjusted to this electronic calendar. [CG012]* *Sometimes in the morning*,* she does not prepare (breakfast)*,* I will go to the hotel and bring…I’m least bothered about all these things. She took 15–20 min extra; it doesn’t matter to me. We can adjust it. [CG010]* *If it was given earlier*,* it would have been better to her [CG004]* *I mean*,* I might use a book whereas someone else might use their mobile*,* so it becomes very subjective to each individual. We are now imposing a medium saying*,* oh*,* you need to look at this board. And that’s something that might be alien to the person…Maybe you can have an app for those who are happy to use a mobile phone and maybe give guidance on how you can use a book. (CG011)*
Contextual considerations	Living arrangement, attitudes towards devices, elders and time in India	*Here (in India)*,* more than gadgets*,* the personal touch is influencing the person. [CG004]* *It will depend on patient to patient. Mother would have used it maybe if she was living alone. Maybe she would have used it more often. Since all of us are there she can keep herself engaged. Because such an environment is different. So maybe that matters. [CG009]* *Here (in India) we take things for granted we can do at any time. [CG004]* *But previously she was very dutiful. If she’s having some important work or role to do*,* then these things (assistive devices) are important. [CG010]* *Especially with the person getting aged they will never leave their old habits*,* it’s very difficult to convince them to adapt to a certain change. okay. And modern gadgets with the aged people*,* it very difficult to convince. [CG004]* *I mean*,* at this age it’s not easy for*,* I think*,* anybody to*,* you know*,* learn. Especially if this is an electronic thing. It’s a totally new domain. [CG008]* *The use of a clock has been for time but anything further than that is alien for the majority of the population (in India)*,* especially the elderly of today. But however*,* ten years down the line it’s a different concept. When the younger generation becomes older*,* the acceptance for these devices becomes higher. That will happen in less than a decade. So right now*,* if you wish to implement this device*,* then yes*,* in certain areas the level of education will have a different impact. You will have to look at it from that perspective. [CG011]*

### Motives

The motivation of PwD and caregivers to use devices needs to be considered. PwD should feel the need for the device, which would depend on their awareness of their abilities or deficits, present DTM strategies, daily routine or responsibilities. Prior to the TMP provision, PwD relied mostly on caregivers and established routines for DTM. Some PwD use everyday products such as radio, newspapers, wall calendars, and alarm clocks for DTM. Some also used smartphone features such as reminders and social media apps such as Facebook and WhatsApp. They were comfortable with their familiar routines and ‘regular’ products and were not very keen on using a new product for their DTM.

P001, a housewife with mild dementia was able to engage in household chores. She experienced difficulties in DTM and was motivated to use the device to keep track of time. However, other PwD, especially older men, did not have any major tasks or responsibilities and did not feel the need to do things on time. The COVID-19 pandemic was another factor that affected the routines of PwD and may have affected their need for devices.

*It (time management) was important before. However*,* not now. There’s nothing now. I wake up*,* that is there. Other than that*,* there’s nothing. Actually*,* I do not truly do anything. [P001]*

*Since the lockdown*,* all his routine has completely changed*,* and most of the time*,* he is idle not knowing what to do. That had a negative effect on him. [CG003]*

Most caregivers expressed their motivation to try anything that would support PwD’s memory or just confidence in knowing the time or doing valued activities. However, some felt that the PwD need not do any work or do not have any important responsibilities and need not be punctual. CG010 said,

*Time and all she is not following. No need also. I will tell you again…She’s not dependent on time. She’s purely a homemaker. In addition*,* she’s not having any priority*.

For some caregivers, such as CG001, increasing the independence of the PwD to reduce caregiving demands was a motivation for using the device. He found the device helpful and was willing to purchase the same device.

*Yes*,* I would buy it. I got to know about these after you gave it. If it was available and she needed it*,* then I would buy it… If she improves*,* it is possible to go out. Otherwise*,* the problem is that*,* along with other things*,* we will have to look out for her too.*

The devices were given free-of-cost in the study, which may have supported their motivation.

### Personal characteristics

PwD and caregivers’ comfort, past experiences and familiarity with technology, personality traits, and willingness to try new things may impact device adoption and use. In addition, the cognitive abilities and preferences of PwD may need to be considered..

*I mean*,* I might use a book whereas someone else might use their mobile*,* so it becomes very subjective to each individual. We are now imposing a medium saying*,* oh*,* you need to look at this board. In addition*,* that is something that might be alien to the person… Maybe you can have an app for those who are happy to use a mobile phone and may provide guidance on how you can use a book. (CG011)*

Additional caregiver commitments, such as employment and/or other caregiving roles, may also influence device use. CG001 was working, and his wife was also a primary caregiver for her mother: *‘However*,* the thing is that even I am not able to make time for it’.* The caregiver’s attitudes towards the PwD are important considerations that may depend on the nature and quality of their relationship. This, in turn, may be influenced by their personal characteristics, such as age, gender, education, and past or current occupations. For example, in our study, the adult children or children-in-law and wives of PwD appeared to be more willing to try the devices and support the PwD’s DTM. However, CG010, a husband of PwD001 with mild dementia, appeared to be uninterested in supporting his wife’s motivation to perform valued occupations in a timely manner.

*Sometimes in the morning*,* she does not prepare (breakfast)*,* I will go to the hotel and bring…I’m least bothered about all these things. She took 15–20 min extra; it does not matter to me. We can adjust it. [CG010].*

Additionally, women PwD, including those who had retired from paid employment and self-employed men (e.g., business), appeared to be engaged in some tasks and used devices for DTM. However, retired men appeared to have limited activities in addition to self-care. They may have a limited need for a DTM and thus devices.

### Contextual considerations

The participants’ narratives suggest the influence of living arrangements and sociocultural attitudes on the adoption of TMP. The participants opined that devices may be suitable for older adults living alone. Older adults living with family members may not need such devices, as they have the help of family members. The participants felt that people in India prefer cues from others, especially family members, rather than devices. Some participants expressed that with advancing age, it is difficult for older people to learn new things or change. CG010 also felt that time management was important only for those who had important work or were employed. Others, especially homemakers such as his wife, need not be punctual and would not need such devices.

*However*,* previously she was very dutiful… If she’s having some important work or role to do*,* then these things (assistive devices) are important. [CG010]*

The participants also felt that doing things in time was not important for people in India in general. CG004 said, *‘Here (in India)*,* we take things for granted*,* we can do at any time’.* One caregiver envisaged changes in these attitudes among younger cohorts and recommended initiatives to address their needs in the near future.

*The use of a clock has been for time but anything further than that is alien for the majority of the population (in India)*,* especially the elderly of today. However*,* ten years down the line*,* it is a different concept. When the younger generation becomes older*,* the acceptance of these devices becomes greater. (CG011)*

## Discussion

The results of the pre-post study indicate that the TMP helped maintain or marginally improve PwDs’ TPA, performance and satisfaction in valued daily activities and well-being. However, their Time-S-Senior scores for subjective DTM appeared to have decreased significantly, especially in PwD with moderate dementia. This could be attributed to a reduced sense of control over their DTM due to disrupted routines during the COVID-19 pandemic. Nevertheless, the caregiver scores on Time-P show that PwD were able to maintain their DTM at the posttest. While the caregiver-related outcome measures did not change significantly at posttest, they showed declining trends. In addition, the effort required by caregivers to support device use by PwD, caregiver well-being and coping could be influenced by factors such as other responsibilities and the pandemic. Overall, the pre-post study indicated that TMP helped in maintaining functional abilities and well-being, especially of PwD with mild dementia, and maintaining caregivers’ coping ability and well-being to some extent. These findings are consistent with the qualitative component of the study. In addition to the maintenance of PwDs’ TPA and DTM, the qualitative study revealed other benefits of TMP such as reduced annoyance with caregivers’ prompting, improved confidence in knowing the time and doing valued activities more independently. These subjective benefits that support the self-efficacy of the PwD are as important as cognition or functional independence. Studies in developed countries on TMP have also reported limited to reasonable success with time aid interventions [10, 12, 16, 17, 25].

For DTM, the participants appreciated TMP supporting time orientation and providing reminders. Advanced devices such as electronic planners and planning aids were also appreciated but required the caregivers to be comfortable with technology and put considerable effort into doing so. The findings are similar to those of studies from developed countries that used these TMPs [10, 12, 16, 19]. Although simple and portable, timers were not preferred, as the duration of the activity may not be important for PwD or caregivers. Additionally, PwD may not remember to use timers for an activity. The participants did not prefer local products. The visual display of the standard digital clock was difficult to read. The pictorial planners were not preferred, as they appeared childish and seemed to draw untoward attention from visitors [36].

In addition to age-related considerations of vision and hearing, the participants provided several suggestions to make the devices suitable for the Indian context. The constant need for electricity for the TMP was reported as a concern in the present study. Similarly, a multicountry study in Europe reported that PwD unplug devices due to concerns over electricity [16]. The caregivers’ suggestion in the present study to use battery-operated devices is a practical suggestion considering frequent power cuts, multiple users in joint families and limited electrical outlets at homes in the study setting and probably in other regions of India. The size and placement of the device appeared to be a concern. The physical environment, such as placement, availability of space and electrical outlets, also needs to be considered. As seen in the present study, the devices that were placed in locations with easy access and used by the family, supported device used by the PwD. Portability may also be another desirable important feature, as it is very common for Indian older adults to relocate frequently to reside with their children and relatives for various durations. The easy-to-use electronic calendar for time orientation was the most preferred product, which is in line with the expectations identified in our previous study [36]. The participants suggested adding features to the same product to support DTM. Recent products [24, 54] meet these suggestions and may need to be explored in the Indian context.

The study also elucidated personal, social and cultural factors that need to be considered in the provision of TMP. In line with our previous study [36], PwD and caregiver characteristics such as education, premorbid and preretirement employment, and personality traits, including degree of flexibility and familiarity with technology, were identified as salient factors. In addition, this study highlighted the importance of understanding the motives of PwD and caregivers for using TMP, which is consistent with studies from developed countries [10, 12, 16, 19].

As seen in the present study, the motives of the PwDs to use TMP could be just the confidence of knowing the time and sense of control, being able to read the time, or being able to perform valued daily activities with fewer cues [10, 12, 19]. PwDs’ attitudes towards time are an important factor influencing their motivation to use TMP. Following the onset of dementia, PwDs’ attitudes towards time may change due to the neurobehavioural symptoms associated with the condition, thereby influencing acceptance of TMP [10]. The present study provided further insights into contextual factors that may also influence PwD’s attitudes towards time and motivation to use TMP. In India, DTM, especially the timing and duration of an activity, may not be valued by PwD and caregivers because of the prevalent polychronic attitudes towards time [36]. PwD mostly rely on caregivers and established routines for DTM [10, 12]. Some PwD used everyday products such as diaries, calendars, newspapers, radio or mobile phones for DTM and may not be motivated to use new unfamiliar products imported for the study [10], especially products that make them look or feel different from others [10, 26, 36]. They did not favour the readily available and easy-to-see whiteboards, which are common time management tools, especially in developed countries. Nonacceptance of TMP by PwD due to stigma has been reported in previous studies [26] but may be more pronounced in countries such as India, which has deep-seated prevalent negative attitudes towards people with disabilities, older people, and devices [36]. With disease progression, PwD may be unable to use everyday products and may also have difficulty learning to use TMP [25, 55]. Encouraging the use of TMP in the early stages of dementia may be beneficial [10, 12, 17, 25, 36].

Culturally prevalent norms and practices may also influence the motivation for DTM and thus TMP. In the present study, women (homemakers or retired) and self-employed men were involved in some productive work, whether household chores or business, albeit with caregivers’ support. However, retired men were not engaged in any meaningful activities and therefore did not feel the need for DTM and TMP. Following retirement, they may have had difficulty filling their free time with leisure or work due to limited resources, opportunities or personal choices. Culturally, views on retirement in India revolve around withdrawing socially and choosing a spiritual path [56]. In general, retirees in India are comfortable with ‘free’ time in their hands for rest and relaxation [57, 58]. They also feel that retirement is the time to rest and expect their children to look after them [59]. However, compared with retired men, older women spend more time in household chores because of socially ascribed gender-based norms that cut across age groups and socioeconomic status [58, 60, 61]. Retired men are often reluctant to participate in household chores that are considered women’s responsibilities [62, 63]. The present study findings also elucidate gender-based stereotypes toward women, especially by husbands, that are prevalent in patriarchial societies such as India [64]. These socially acceptable prejudiced attitudes may deprive older women with dementia of access to TMP and other rehabilitation services.

Family members also tend to take over older persons’ responsibilities, especially the more complex instrumental activities of daily living. These ‘caring’ attitudes towards older adults stem from filial piety and interdependence, which are common in the collective culture prevalent in Indian society. It is thus acceptable for older adults to receive help and for adult children to provide help [65]. These attitudes also reflect the prevalent ageist attitudes in Indian society [66], especially benevolent or positive ageism, where older adults are seen as vulnerable and needing help. Such attitudes may limit older adults’ participation in basic activities of daily living [67, 68]. Reduced participation in complex daily activities is known to correlate with cognitive decline in older persons [69]. The onset of dementia further restricts their participation in safety concerns or difficult behaviors [70] and may be compounded by negative attitudes towards PwD [71]. This lack of opportunities to perform meaningful tasks has detrimental consequences for PwD and may exacerbate disease progression. In line with studies in developed countries, the participants in the present study also felt that TMP would be more valued by PwD living alone [10, 19]. In India, joint families are the preferred norm. However, these living arrangements are changing [33] and are reflected in the present study, with several PwD living alone or with their spouse with the support of children or relatives living nearby. With an increasing number of older adults living alone in India, it is possible that, in the future, PwD may be motivated to use TMP to support independent living.

Similarly, caregivers’ motives to use TMP also need to be understood [10, 12, 26]. This would depend on their understanding of dementia, caregiving priorities, and relationships with PwD. In India, awareness of dementia is generally low, and owing to the prevalent medical model of practice, the focus of intervention is usually on client factors to support or improve memory, cognition, or orientation [36]. Devices such as the electronic calendar would be adequate for such motives. Our study also revealed changing trends in caregivers’ attitudes. Younger caregivers were motivated to use the TMP to make PwD more independent and reduce their own supervision and burden. This finding was similar to those of studies from Sweden [10, 12, 26] and was surprising for this study setting, where people are conservative and tend to uphold traditional values and customs of taking over the responsibilities of their parents. Although they are employed and have other caring responsibilities, most caregivers invest considerable time in caregiving. In such cases, providing advanced devices in the initial stages of dementia may be appropriate to support the maximal independence of PwD [10, 12, 16]. Although caregivers did not benefit much with the TMP, they were interested in using the devices, which is consistent with findings from developed countries [12, 26]. It was heartening to see caregivers valuing PwD’s confidence and well-being, as it would support PwD’s quality of life, an implicit objective of dementia care. The study also identified lack of time, support, and device complexity as factors that may deter caregivers in supporting PwD to use TMP, especially older caregivers.

The use of AT by people with cognitive impairements in India is relatively nascent. The present study involved salient stahekolders, PwD and caregivers, whose experiences and perspectives shed light on factors influencing influencing AT implementation and adoption by people with cognitive impairments in the Indian context including facilitators suh as ease of use and cultural relevance of devices, and barriers such as lack of knowledge about aging, dementia and AT, limited access, cultural attitudes and practices. The use of AT requires a systemic approach that addresses all aspects such as AT development, provision, access, support and maintenance [72]. The present study points to the obvious absence of such a system to support AT adoption for cognitive impairments in India and elucidates an urgent need for the same to address the needs of the aging population. These findings are consistent with a recent systematic review on adoption and implementation of AT worldwide [73].

### Strengths, limitations and recommendations

The study’s strength is its study design. The use of mixed methods supported methodological triangulation. The outcome measures for the quantitative component were adapted and validated for the Indian context. KB completed the training and certification for the KaTid-Senior and CDR. The recommended guidelines were used for all adaptations and translations. Several measures were used to support the trustworthiness of the qualitative component of the study. We did triangulation by source of data (PwD and caregivers) and investigators (multiple researchers with varying professional and clinical expertise). The inclusion of both PwD and caregivers enabled a better understanding of the use of TMP in the Indian context. The PwD and caregivers were interviewed separately, enabling open expression without the influence of the other. To support transferability, detailed descriptions of the study setting and participants were provided. Maximum variation sampling was performed with respect to living arrangements, financial status, and the relationships between PwD and caregivers. The use of reflexivity and peer debriefing throughout the study ensured the confirmability of the findings. Dependability is supported by a detailed description of the methods and the involvement of experienced and knowledgeable researchers with a good understanding of the context.

The COVID-19 pandemic necessitated telephone interviews in addition to face-to-face interviews. While all the measures used to address this issue, such as the use of additional probes and the scheduling of interviews in a distraction-free environment [74], we cannot rule out data loss in the absence of observational data. The pandemic also had deleterious consequences for healthy individuals and otherwise across all age groups, including PwD and caregivers [75–77], which may have impacted the participants’ involvement in the study. The findings need to be used with caution in the absence of a control group and the small sample size of PwD. Additionally, the participant characteristics are not representative of all geographic regions, socioeconomic statuses, or cultures in India.

The findings justify future studies with more robust designs, such as randomized controlled trials in different settings and longer follow-ups. Research on developing contextually relevant products should prioritize the changing demographics, attitudes, and emerging needs of the aging population.

## Conclusion

The findings suggest that although DTM may not be a priority in India due to the prevalent polychronic attitudes and collective culture, PwD and caregivers may be open to the use of TMP for its subjective or intangible (e.g., self-efficacy, wellbeing, satisfaction, coping) and/or objective (cognition, independence, burden) benefits. The study recommends a person-centred, non-stigmatizing, context-specific, and culturally appropriate approach to AT implementation and adoption for PwD. Engagement in meaningful activities and supportive routines is an essential component of dementia care that also slows disease progression. However, the prevalent sociocultural influences and discriminatory attitudes inadvertently abett idleness and dependence among PwD and add to caregiver burden. Dementia education and interventions should be tailored to address these attitudes, encourage maximal participation in the daily activities of PwD with rehabilitation interventions such as AT and reduce caregiver burden. The findings justify the approaches to TMP provision proposed in our previous study: the DTM-oriented approach, DTM + education, the impairment-oriented approach, and respect for personal preferences [36]. Educating and involving all family members may also support the adoption of TMP by PwD, especially in joint families [36]. The study findings can inform AT implementation and adoption for people with cognitive impairments in the Indian context. The study provides impetus to intitiatives for improving access to AT in LMICs [78] and supporting the well-being of PwD and caregivers [79].

## Data Availability

No datasets were generated or analysed during the current study.

## Abbreviations

LMIC: Low- and middle-income countries
PwD: Person with Dementia
TPA: Time Processing Abilities
DTM: Daily Time Management
AT: Assistive Technology
LMIC: Low- and middle-income Countries
KaTid-Senior: Kit for Time-processing Ability-Senior
Time-S-Senior: Time-Self rating Senior
Time-P-Senior: Time-Proxy Senior
COPM: Canadian Occupational Performance Measure
WHO-5: World Health Organization-5 Well-being Index
COPE Index: Carers of Older People in Europe Index
TMP: Time Management Products
